# Five-fraction CyberKnife radiotherapy for large brain metastases in critical areas: impact on the surrounding brain volumes circumscribed with a single dose equivalent of 14 Gy (V14) to avoid radiation necrosis

**DOI:** 10.1093/jrr/rrt127

**Published:** 2013-11-01

**Authors:** Hiroshi K. Inoue, Hiro Sato, Ken-ichi Seto, Kota Torikai, Yoshiyuki Suzuki, Jun-ichi Saitoh, Shin-ei Noda, Takashi Nakano

**Affiliations:** 1 Cyber Center, Kanto Neurosurgical Hospital, 1120 Dai, Kumagaya, Saitama, 360-0804, Japan; 2 Gunma University Heavy-ion Medical Research Center, 3-39-22 Showa-machi, Maebashi, Gunma, 371-8511, Japan; 3 Department of Radiation Oncology, Gunma University Graduate School of Medicine, 3-39-22 Showa-machi, Maebashi, Gunma, 371-8511, Japan

**Keywords:** large brain metastases, hypofractionated radiotherapy, five-session radiosurgery, radiation necrosis, V14

## Abstract

The efficacy and toxicity of five-fraction CyberKnife radiotherapy were evaluated in patients with large brain metastases in critical areas. A total of 85 metastases in 78 patients, including tumors >30 cm^3^ (4 cm in diameter) were treated with five-fraction CyberKnife radiotherapy with a median marginal dose of 31 Gy at a median prescribed isodose of 58%. Changes in the neurological manifestations, local tumor control, and adverse effects were investigated after treatment. The surrounding brain volumes circumscribed with 28.8 Gy (single dose equivalent to 14 Gy: V14) were measured to evaluate the risk of radiation necrosis. Neurological manifestations, such as motor weakness, visual disturbances and aphasia improved in 28 of 55 patients (50.9%). Local tumor control was obtained in 79 of 85 metastases (92.9%) during a median follow-up of eight months. Symptomatic edema occurred in 10 patients, and two of them (2.6%) required surgical resection because of radiation necrosis. The V14 of these patients was 3.0–19.7 cm^3^. There were 16 lesions with a V14 of ≥7.0 cm^3^, and two of these lesions developed extensive brain edema due to radiation necrosis. None of the patients with a V14 of <7.0 cm^3^ exhibited edema requiring surgical intervention. We therefore conclude that a high rate of local tumor control and low rates of complications can be obtained after five-fraction CyberKnife radiotherapy for large metastases in critical areas. The V14 of the surrounding brain is therefore a useful indicator for the risk of radiation necrosis in patients with large metastases.

## INTRODUCTION

The majority of brain metastases occur in patients with advanced stages of primary cancer, and brain metastases may decrease the patient's quality of remaining life, because symptoms such as hemiparesis, aphasia, hemianopia, dementia and disturbances of consciousness occur, especially in patients with large brain metastases in critical areas, including the brainstem. The survival period of patients with untreated brain metastases is reportedly one to three months [1]. The radiation therapy oncology group–recursive partitioning analysis (RTOG–RPA) of prognostic factors has shown that the best survival (median: 7.1 months) is of patients in Class 1: < 65 years of age with a Karnofsky performance status (KPS) of ≥ 70, and a controlled primary cancer with the brain demonstrating only metastases. In contrast, the worst survival (median: 2.3 months) is found in Class 3 patients, with a KPS of < 70 [2]. The KPS is especially low in patients with a dysfunction in critical areas, such as the motor cortex, visual pathways and brainstem. The optimal treatment of large brain metastases in these areas may contribute to improving the KPS and quality of remaining life for such advanced cancer patients. The therapeutic approaches for brain metastases include surgery, whole-brain radiotherapy (WBRT), radiosurgery and chemotherapy. Many patients are treated with a combination of these approaches, depending on the clinical stage of the primary lesion and the number, size and site of the brain metastases. However, treating large brain metastases in critical areas effectively is not easy [3]. Surgery has the risks of inducing a functional deterioration, and single-fraction radiosurgery has dose limitations for the surrounding critical brain areas. WBRT and chemotherapy are usually not able to control large brain metastases. Fractionation or multisession radiosurgery is an option for treating large brain metastases in critical areas that can help to reduce the adverse effects on surrounding structures, as reported for the treatment of gliomas and perioptic lesions [4–5]. However, the optimal number of fractions, the marginal isodose (%) and the marginal dose have not been established, and the exact incidence of adverse effects on the surrounding brain is unclear. Morbidity and even mortality have been reported after fractionated radiosurgery for large brain metastases [6–7]. We have previously reported a study of three-fraction radiotherapy for brain metastases in critical areas, including larger tumors, and recommended that larger fraction numbers should be selected for large brain metastases [8]. In this study, five-fraction radiotherapy was intended to treat large brain metastases in critical areas to avoid causing any dysfunction of the surrounding brain and to maintain sufficient treatment doses for malignant lesions.

This report presents the efficacy and toxicity of five-fraction CyberKnife radiotherapy performed at this institution as a useful treatment option for large brain metastases in critical areas, including tumors > 30 cm^3^ (4 cm in diameter).

## MATERIALS AND METHODS

All patients gave their written informed consent prior to the procedure, and 1016 patients with brain metastases were treated with single-fraction radiosurgery or hypofractionated radiotherapy at the Kanto Neurosurgical Hospital between March 2005 and March 2013. Hypofractionated treatment was performed for 392 patients. This report analyzed a consecutive series of five-fraction treatment for large lesions in critical areas, and followed these 78 patients (85 brain metastases) received five-fraction treatment and followed for more than six weeks with imaging studies for 85 brain metastases, including tumors > 30 cm^3^. The median age of the patients was 65 years old, and 39 patients (50.0%) were ≥ 65 years old. The patients' primary cancers were located in the lung (*n* = 31), breast (*n* = 16), gastrointestinal tract (*n* = 13), kidney (*n* = 4), uterus (*n* = 3), ovary (*n* = 2) and other regions (liver, testis, larynx, bladder, etc.). Of the 78 patients, 41 (52.6%) had metastases to other organs and 43 patients (55.1%) had multiple brain metastases (**Table 1**). The tumors treated with the five-fraction protocol were situated in the frontal lobe (close to the optic pathway, Broca's area and the motor cortex), parietal lobe (sensory cortex and dominant angular cortex), temporal lobe (close to the optic pathway and Wernicke's area), occipital lobe (visual cortex), thalamus, basal ganglia, brainstem or cerebellum close to the brainstem. WBRT or single session radiosurgery had previously been administered to 10 patients (12.8%). Of the 78 patients, 55 (70.5%) had neurological manifestations due to the lesions that we intended to treat using five-fraction radiotherapy. The neurological manifestations observed before treatment included motor weakness in 28 patients (35.9%), unsteady gait in 12, visual disturbances in 9, aphasia in 5, numbness in 6, agraphia in 3, urinary incontinence in 2, and focal seizures and dementia in 1 patient each. The KPS of 44 patients (56.4%) was < 70, and all patients were in RTOG–RPA Class 2 or 3. The initial tumor volume was measured using the MultiPlan (Accuray, Sunnyvale, CA) software program, which determines the treatment volume based on enhanced T1-weighted magnetic resonance imaging (MRI). The median tumor volume was 12.6 cm^3^ (up to 45 cm^3^). There were 32 tumors > 15 cm^3^ ( > 3 cm in diameter), but 9 tumors in the brainstem were < 10 cm^3^. **Table 1** shows the patient characteristics.

**Table 1.** Characteristics of patients treated with five-fraction CyberKnife radiotherapy

Number of patients	78	Location of tumor	
Median age (range)	65 (37–85)	Cerebral hemisphere	57
Age ≥ 65	39	Cerebellum	14
Age < 65	39	Brainstem	14
Sex		Neurological manifestations	in 55 patients
Male	43	Motor weakness	28
Female	35	Unsteady gait	12
Primary cancer		Visual disturbances	9
Lung	31	Aphasia	5
Breast	16	Numbness	4
Gastro-intestinal tract	13	Others	7
Kidney	4	Median KPS score	60 (40–90)
Uterus	3	KPS ≥ 70	34
Ovary	2	KPS < 70	44
Others	9	Tumor volume, median (cm^3^)	12.6
Multiple vs single		>15.0	32
Multiple metastases	43	10.0–15.0	23
Single metastasis	35	<10.0	30
Metastases to other organ	41	Imaging follow-up period (months) (range)	8 (2–42)
Previous radiotherapy	10	Survival period (months) (range)	8 (2–45)

### Five-fraction radiotherapy

All patients evaluated in this study were treated consecutively with five-fraction radiotherapy over five sequential days (daily treatment) using a CyberKnife (Accuray, Sunnyvale, CA). Patients with perifocal brain edema and progressing symptoms were treated with the concomitant intravenous administration of glycerol and beta-methasone (osmo–steroid therapy). All treatment procedures were performed under computed tomography (CT) and MRI (1.5-T or 3.0-T) guidance in a frameless system. Critical areas, such as the optic pathway, brainstem and other cranial nerves, were identified using CISS (heavy T2) images (MR cisternography). The gross tumor volume (GTV) was demarcated for the enhanced lesions from fusion images of enhanced CT and MR using 1 mm thick axial images. The clinical target volume (CTV) was identical to the GTV (CTV = GTV) for treatment planning to measure the exact surrounding brain volume within the isodose line. More than 90 or 95% of the target volume was intended to be covered with the same 50–70% isodose line as single-fraction radiosurgery, instead of the 80–90% isodose line typically used in hypofractionated radiotherapy. The marginal dose was intended to be 31–35 Gy at the prescribed isodose line, and intended to cover more than 90 or 95% of the target, depending on the tumor volume and surrounding critical structures. The maximum dose to the optic pathway (optic nerve, chiasm and tract) was intended to be < 15.6 Gy, rather than 25 Gy (the normal tissue dose constraint for the optic pathway in five-fraction treatment, an equivalent dose of 8 Gy in a single fraction treatment) [9] to reduce the risk of adverse effects.

### Evaluation of the brain volume around lesions involved in the isodose line

The isodose volume of the surrounding brain (excluding the GTV) circumscribed with a 28.8 Gy dose line was measured and recorded in each patient to determine the risk of adverse effects on the surrounding brain. The 28.8 Gy dose (instead of 28 Gy or 30.0 Gy, the normal tissue dose constraints for the spinal cord and cauda equina, respectively, in five-fraction treatment), an equivalent dose of 14 Gy in single-fraction treatment [9], was used to compare and integrate the findings with other hypofractionated treatments [8, 10]. The V14 (surrounding brain volume circumscribed with a single dose equivalent of 14 Gy), as well as tumor volumes included within the prescribed marginal isodose line, were calculated from the integral dose–volume histograms (DVHs) as reported previously [8], or were measured using the MultiPlan software program for the G4 system (Accuray, Sunnyvale, CA). The V14 of each patient was evaluated in relation to the toxicity (brain edema and necrosis) to the surrounding brain and critical areas, such as the brainstem and cerebellum close to the brainstem.

### Follow-up evaluations and patient data

Changes in the neurological symptoms, such as paresis, sensory disturbances, aphasia and visual disturbances, were examined after treatment in patients with large metastases in critical areas causing related symptoms. The severity of symptoms was divided into four grades based on the activities of daily living determined using the medical care accreditation criteria: Grade 0 = no trouble (able to perform the activities without help), Grade 1 = slightly impaired (able to do the activities with some difficulty), Grade 2 = moderately affected (needing partial support), and Grade 3 = severely affected (unable to function in normal daily life and needing total support). The improvement of symptoms was defined as an increase by one grade or more.

Serial imaging studies (MRI or CT) with thin sections (1–2 mm thickness) were requested six weeks after treatment and every two to three months thereafter. Patients who lived far from the center were examined by their referring physicians. Contrast-enhanced imaging studies were used to define the tumor response and local control. The tumor volumes were calculated using the geometric method using the diameter of three dimensions (*x*, *y* and *z*) of the ellipse obtained from axial and coronal slices of serial imaging studies [8, 11]. The calculated volume was within a 15% error of the volume data obtained using the MultiPlan software. The tumor response was then divided into four groups: almost disappeared (tumor volume decreased >95%), reduced (tumor volume decreased 15–95%), stable (tumor volume change within ±15%), and enlarged (tumor volume increased >15%) to compare the response with former reports for larger brain metastases [3, 8]. The incidence of brain edema and necrosis was examined in relation to the V14 of the surrounding brain.

### Statistical analysis

The differences between the groups were evaluated using Student's *t*-test. The cumulative incidence was estimated according to the Kaplan–Meier method, and was examined for significance with a log-rank test and a generalized Wilcoxon test. All analyses used the conventional *P* < 0.05 level of significance*.*

## RESULTS

The prescribed isodoses ranged from 50–76% (median, 58%) for the target. The marginal dose ranged from 25–40 Gy (median, 31 Gy), and the maximum dose ranged from 39.5–67.8 Gy (median, 55.4 Gy) delivered in five fractions. Osmo–steroid therapy was administered to 16 patients during the five-fraction CyberKnife radiotherapy for symptoms due to perifocal edema.

### Follow-up evaluations

The neurological manifestations observed in 55 patients before treatment improved in 28 of the patients, beginning several weeks to several months after treatment, and were accompanied by a tumor response. Motor weakness was improved one grade or more in 16 patients. Three of the seven patients with Grade 2 paresis (requiring support for everyday activities) recovered to have almost normal function, and four patients improved to Grade 1 (not requiring support). All five patients with Grade 1 paresis recovered to normal function. Aphasia improved in four patients, unsteady gait improved in six, visual disturbances in four, numbness in two, and dysarthria, incontinence and dementia improved in one patient each (**Table 2**). The KPS improved in 16.7% of the patients after treatment as a result of neurological improvements that changed their performance status. Three patients were able to return to their workplace, and two patients were able to resume their work as housewives (KPS: 90). There were no new neurological deficits from direct damage to the brainstem or functional areas, although symptoms recurred or appeared in 10 patients due to adverse effects (brain edema and necrosis) on the surrounding brain.

**Table 2.** Results of five-fraction CyberKnife radiotherapy

Median prescribed isodose (range)	58 (50–76) %
Median marginal dose (range)	31 (25–40) Gy
Improved neurological manifestations (no. of Grade 0, 1, 2 and 3 cases)	28/55
Motor weakness	16/28
before radiotherapy	(0, 5, 7, 4)
after radiotherapy	(8, 4, 4, 0)
Unsteady gait	6/12
before radiotherapy	(0, 3, 3, 0)
after radiotherapy	(3, 3, 0, 0)
Visual disturbances	4/9
before radiotherapy	(0, 4, 0, 0)
after radiotherapy	(4, 0, 0, 0)
Aphasia	4/5
before radiotherapy	(0, 2, 2, 0)
after radiotherapy	(3, 1, 0, 0)
Numbness	2/4
before radiotherapy	(0, 2, 0, 0)
after radiotherapy	(2, 0, 0, 0)
Others	3/7
before radiotherapy	(0, 2, 1, 0)
after radiotherapy	(2, 1, 0, 0)
Tumor response (*n* = 85)	
Almost disappeared (volume decrease >95%)	3
Reduced (volume decrease 15–95%)	78
Stable (volume change ±15%)	1
Enlarged (volume increase >15%)	3 (cyst expansion)
Tumor recurrence	3/85 (3.5%)
Adverse effects	
Radiation edema	8 (newly developed, 4)
Radiation necrosis	2

Grade 0 = no trouble (able to do daily activities without help), Grade 1 = slightly impaired (able to do daily activities with some difficulty), Grade 2 = moderately affected (needing partial support), Grade 3 = severely affected (needing total support).

### Tumor response and local control after treatment

**Fig. 1.**
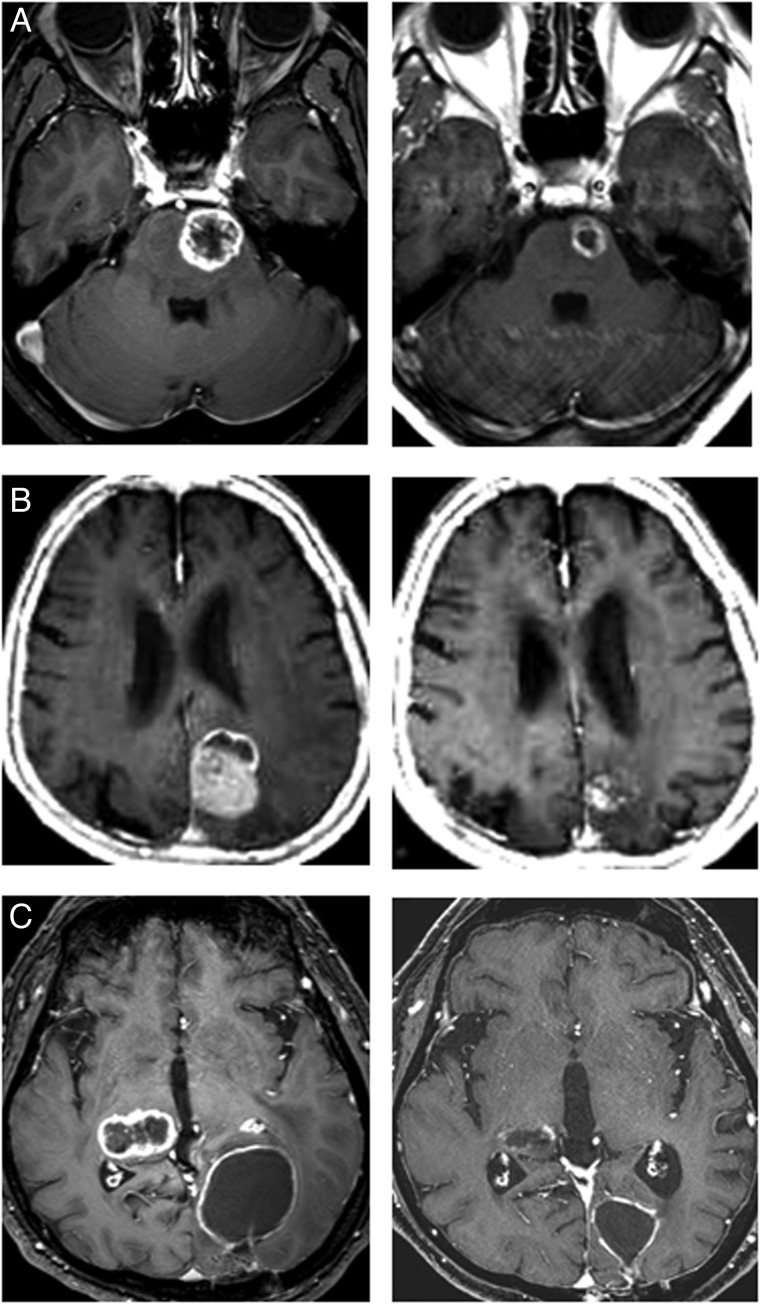
Gd-enhanced T1-weighted MR images. (**A**) Cecal cancer brain metastases in a 43-year-old male. A pons tumor was treated with a marginal dose of 31 Gy in five fractions at a 51% isodose (left). A significant tumor response and no adverse imaging effects were found five months after the five-fraction CyberKnife radiotherapy (right). The right hemiparesis disappeared and the KPS improved from 60 to 70. (**B**) A lung cancer (non-small-cell) brain metastasis in a 78-year-old male. A tumor in the occipital lobe with perifocal edema was treated with a marginal dose of 31 Gy in five fractions at a 54% isodose (left). A tumor response was found eight months after five-fraction radiotherapy (right). (**C**) Lung cancer (non-small-cell) brain metastases in a 53-year-old male. A tumor in the visual area with perifocal edema was treated with a marginal dose of 29 Gy in five fractions at a 53% isodose, and a right thalamic tumor was treated with a marginal dose of 27 Gy in three fractions at a 54% isodose (left). A tumor response was found seven months after treatment. Both tumors decreased in size and no adverse effects were observed (right).

**Fig. 2.**
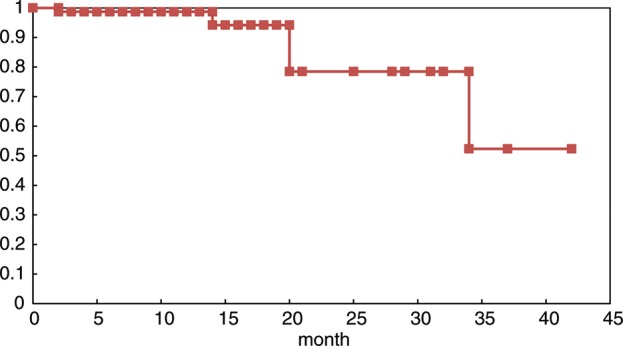
The local control rate of 85 large brain metastases treated with five-fraction radiotherapy. The control rate decreased to 78.5% at two years and 52.3% at three years after treatment because of marginal recurrences.

**Fig. 3.**
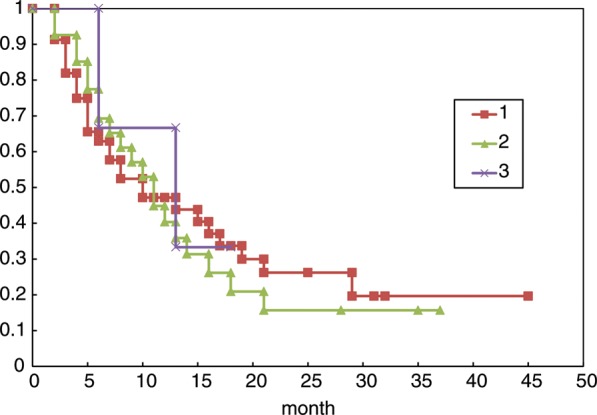
Kaplan–Meier survival curves of patients with large brain metastases in three groups: (i) patients with tumors <15 cm^3^, (ii) patients with tumors 15–30 cm^3^, (iii) patients with tumors >30 cm^3^. No statistically significant differences were found among the groups.

All 85 lesions in 78 patients were subjected to sequential imaging studies from six weeks to 42 months (median, eight months) after treatment. All but four of the 85 lesions (one stable and three enlarged due to cyst expansion) showed tumor regression on follow-up images, including those that were > 30 cm^3^ (4 cm in diameter). (**Fig. 1 A–C**). Three lesions treated with a marginal dose of 31–32 Gy showed marginal recurrence 14–34 months after radiotherapy and required additional treatment. The second treatment was performed only for the recurrent areas, excluding the central areas treated with higher doses. The local tumor control rate was 92.9%, with a median survival of eight months. However, the control rate decreased in long-term survivors (**Fig. 2**). There were no significant differences in the survival rates in patients with tumors <15 cm^3^, 15–30 cm^3^ or >30 cm^3^ after five-fraction CyberKnife radiotherapy (**Fig. 3**).

### Adverse effects (brain edema and necrosis)

Nine patients had recurrent symptoms and one patient had new symptoms because of extensive brain edema requiring osmo–steroid therapy. Eight of these patients showed both clinical and radiological deterioration six weeks to nine months after treatment; however, the symptoms and edema rapidly improved after osmo–steroid therapy. Four of these patients showed newly developed brain edema, and the remaining four patients showed extensions of pre-existing brain edema that had been present before treatment. They received further oral administration of steroids at the outpatient clinic. Only two patients (2.6%) showed symptoms from 5–11 months after treatment, and these two patients required surgical resection because osmo–steroid therapy insufficiently reduced the symptoms. Surgical specimens confirmed the presence of radiation necrosis. The extensive edema rapidly decreased and almost disappeared within four weeks after surgery. No symptomatic adverse effects occurred in patients with either brainstem metastases and/or patients with metastases close to the optic pathway.

### The V14 of the surrounding brain and adverse effects

**Fig. 4.**
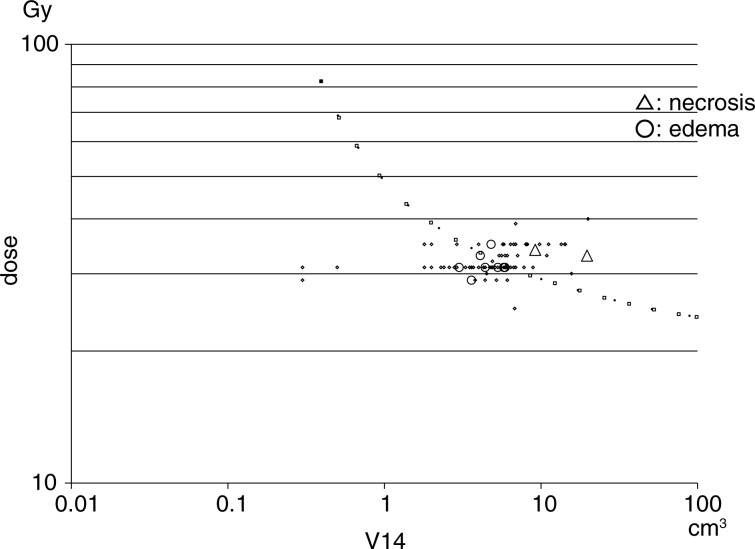
The risk evaluation of adverse effects (brain edema and radiation necrosis). The V14 of 85 brain metastases were plotted in relation to the marginal doses administered during five-fraction CyberKnife radiotherapy. Symptomatic brain edema developed in eight patients (circles) and radiation necrosis requiring surgical resection appeared in two patients (triangles). Kjellberg's 5% necrosis risk line [12] was converted and then drawn for five-fraction radiotherapy (dotted line).

**Fig. 5.**
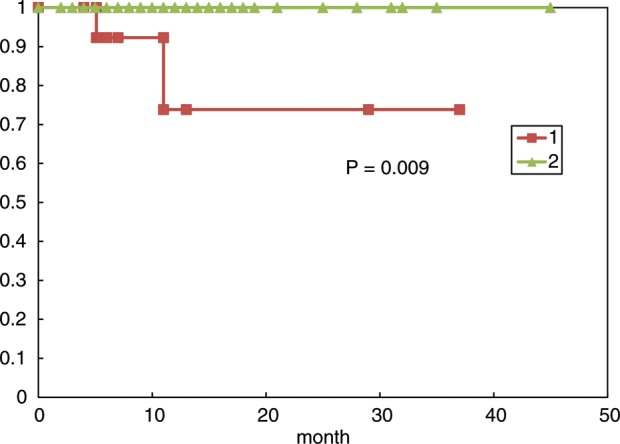
The necrosis-free rate of patients with large brain metastases treated with five-fraction CyberKnife radiotherapy: (i) patients with a V14 ≥ 7.0 cm^3^, (ii) patients with a V14 < 7.0 cm^3^. None of the patients with a V14 < 7.0 cm^3^ (including those who survived >12 months) developed brain necrosis that required surgery.

The V14 was calculated in all patients and plotted in relation to the marginal dose (**Fig. 4**). The median tumor volume of the brainstem (including the thalamus and basal ganglia) metastases was 7.4 cm^3^, and the median V14 in patients with brainstem metastases was 3.0 cm^3^. No adverse radiation-related effects on the brainstem were observed by imaging (**Table 3)**. The median tumor volume of cerebellar and cerebral metastases was 12.6 cm^3^ and 14.3 cm^3^, respectively, and the median V14 in patients with cerebellar and cerebral metastases was 6.2 cm^3^ and 5.7 cm^3^, respectively. Eight patients with cerebral metastases developed brain edema, and two patients with cerebral metastases had radiation necrosis that required surgical removal. The V14 of the patients with brain edema ranged from 3.0–5.9 cm^3^. However, the V14 of the patients with radiation necrosis ranged from 9.2–19.7 cm^3^. Eight of 71 lesions with a V14 of ≥ 3.0 cm^3^ were associated with the development of symptomatic brain edema that required osmo–steroid therapy; however, no patients with lesions with a V14 of < 3.0 cm^3^ had symptomatic brain edema requiring treatment (**Table 4**). Two of the 16 lesions with a V14 of ≥ 7.0 cm^3^ were associated with the development of radiation necrosis that required an operation after treatment; however, none of the 69 lesions with a V14 of < 7.0 cm^3^, including 23 patients who were followed for > 12 months after treatment, was associated with extensive radiation edema requiring an operation. A statistical analysis of the incidence of symptomatic brain edema using a *t*-test demonstrated a significant difference between the group with a V14 of < 3.0 cm^3^ and the group with a V14 of ≥ 3.0 cm^3^ (*P* = 0.002). The incidence of brain necrosis increased in the long-term survival patients with a V14 of ≥ 7.0 cm^3^, however, none of the patients with a V14 of < 7.0 cm^3^ experienced brain necrosis that required surgical removal (**Fig. 5**).

**Table 3.** The V14 and adverse effects of five-fraction CyberKnife radiotherapy

	Brainstem (14)	Cerebellum (14)	Cerebrum (57)
Median tumor volume (cm^3^) (range)	7.4 (0.5–16.6)	12.6 (5.4–23.0)	14.3 (2.5–45.0)
Median prescribed isodose (%) (range)	57 (51–67)	62 (53–67)	58 (50–76)
Median marginal dose (Gy) (range)	31 (29–35)	31 (31–39)	31 (25–40)
Median maximum dose (Gy) (range)	55.4 (46.3–60.8)	52.4 (46.3–61.4)	55.3 (39.5–67.8)
Median V14 (cm^3^) (range)	3.0 (0.3–6.4)	6.2 (2.6–11.2)	5.7 (1.8–20.0)
Symptomatic brain edema	0/14	0/14	8/57
V14 (cm^3^)			3.0–5.9
Radiation necrosis	0/14	0/14	2/57
V14 (cm^3^)			9.2–19.7

**Table 4.** The incidence of adverse effects and the association with V14

Symptomatic brain edema	V14 < 3.0 cm^3^	V14 ≥ 3.0 cm^3^	*P-*value
Number of lesions	0/14	8/71	0.002
Median tumor volume (cm^3^) (range)	4.7 (0.5–14.9)	14.0 (4.0–45.0)	
Median V14 (cm^3^) (range)	2.2 (0.3–2.9)	5.8 (3.0–20.0)	
Follow-up period (months)	2–21	2–45	
No. survived >12 months	3	24	
Radiation necrosis requiring surgery	V14 < 7.0 cm^3^	V14 ≥ 7.0 cm^3^	*P-*value
Number of lesions	0/69	2/16	0.08
Median tumor volume (cm^3^) (range)	12.4 (0.5–45.0)	19.2 (5.8–37.4)	
Median V14 (cm^3^) (range)	4.9 (0.3–6.9)	10.4 (7.1–19.7)	
Follow-up period (months)	2–45	2–37	
No. survived >12 months	23	4	

## DISCUSSION

This series showed that 56.4% of patients had a KPS of < 70 because of neurological manifestations, which were found in 70.5% of patients. The neurological symptoms improved in 28 of 55 patients. As a result, the KPS in patients with large brain metastases in critical areas improved in 16.7% of the patients after five-fraction treatment. A recovery of motor weakness was found in 57.1% of the 28 affected patients. However, the symptoms caused by large lesions directly involving such areas as the motor cortex, internal capsule, angular cortex and visual cortex persisted even after tumor regression. Five-fraction CyberKnife radiotherapy therefore helps to increase the KPS, at least in patients with symptomatic lesions not directly affecting functional areas, and contributes to improving the daily life quality of patients with large brain metastases.

The role of radiosurgery has been described in 10 institutional studies for patients treated with radiosurgery and WBRT [13]. Radiosurgery also plays a role in the treatment of small multiple brain metastases in advanced cancer patients because of the short treatment time and the absence of the need for general anesthesia. However, the treatment of large metastases in the brainstem and around risky organs has dose limitations for single-fraction radiosurgery. Local tumor control rates ranging from 100% for brainstem metastases have been reported with single-fraction radiosurgery with marginal doses of 13–20 Gy using a Gamma Knife or linear accelerator radiosurgery. The complication rates for brainstem metastases have been reported to range from 0–27% in patients treated with single-fraction radiosurgery [14]. The 12 Gy volume of the brainstem is recommended to be decreased to as low as 0.1 cm^3^ during single-fraction radiosurgery to reduce the occurrence of any adverse effects of radiation on the brainstem, detectable by imaging, and to avoid new neurological deficits [15]. In the current series, 14 brainstem metastases, including thalamus and basal ganglia lesions, were treated by five-fraction radiotherapy. The median tumor volume was 7.4 cm^3^, and the median marginal dose was 31 Gy. All tumors were controlled, and no symptomatic adverse effects on the brainstem were found. The median V14 was 3.0 cm^3^. Five-fraction CyberKnife radiotherapy thus seems to be safe and effective for the treatment of large brainstem metastases.

The five-fraction treatment yielded tumor control rates of 92.9% in patients with large tumors in critical areas. The median marginal dose of 31 Gy at a median prescribed isodose of 58% in five fractions seems to be effective for most large brain metastases, as well as a marginal dose of 20 Gy at the prescribed isodose of 50–60% for small tumors in single-fraction radiosurgery. The median survival of our patients was eight months, and no differences were found in three groups of patients divided by tumor volumes in this series. Tumor recurrence appeared in three patients > 12 months after treatment, and all were from marginal areas of the prescribed isodose. Additional treatment was easily performed for these patients, because the volumes of the recurrence were not large and the risk of radiation necrosis after the second treatment was evaluated to be very low.

Hypofractionation or multisession treatments are used to reduce the incidence of complications and adverse effects on the surrounding brain [5, 16]. The development of new technologies for performing frameless radiosurgery has enabled the treatment of large lesions using multisession treatments or hypofractionation [17–19]. However, the incidence of radiation necrosis is not insignificant for the treatment of large metastases, even when hypofractionation is used [16, 18, 20]. Grade 5 adverse effects may easily appear in patients with large metastases due to a brain hernia resulting from extensive brain edema after treatment [7]. Surgical management after radiosurgery may be required in some patients with progressing symptoms.

The volume involved within the treatment plans is essential, and is widely known as a major factor determining the outcomes of radiosurgery. Large lesions are not suitable for single-fraction treatment, necessitating multiple treatments. The marginal dose is also important for tumor control and protection against adverse effects, including radiation necrosis. A marginal dose of 14–15 Gy is used to treat cavernous sinus lesions and small lesions around the brainstem. However, a marginal dose of 12–14 Gy instead of 15–16 Gy has been suggested in order to avoid complications after radiosurgery for large meningiomas [21–22]. Many data and information about the long-term results of brain metastases, including the risk of radiation necrosis, are available for single-fraction treatment. Adverse effects, including radiation necrosis, have also been shown to occur experimentally in hypofraction treatment, depending on the treatment dose and volume [23]. The volume circumscribed with 14 Gy in single fraction and with a single dose equivalence of 14 Gy during hypofraction (V14) is a good indicator for comparing adverse effects between radiosurgery and hypofraction radiotherapy, as reported previously [8]. We have recommended using a V14 < 7.0 cm^3^ to avoid radiation necrosis that requires surgical management based on the results of our study using three-fraction CyberKnife radiotherapy.

The 28 Gy or 30 Gy typically recommended for the spinal cord or cauda equina is used as a single dose equivalent of 14 Gy in five-fraction body radiotherapy. The 28 Gy of the maximum point dose to the spinal cord, or the 30 Gy volume of the cauda equine < 5 cm^3^, is recommended to avoid complications in these tissues [9]. A 28.8 Gy dose was used as the single dose equivalent of 14 Gy in five-fraction radiotherapy in this study to allow for comparisons with radiosurgery and other hypofractionation strategies. The V14 was calculated in all 85 lesions to evaluate the risk of radiation necrosis, as in three-fraction radiotherapy. The results using 28.8 Gy seemed to be better than those using 30 Gy for estimating the complication risk in five-fraction treatment, and also appeared to be compatible with the results using 23.1 Gy in three-fraction radiotherapy [8]. The exact calculation method that should be used to obtain the proper single dose equivalent of 14 Gy in five-fraction radiotherapy has not yet been established [24]. A generalized linear-quadratic model seems to be suitable for the calculation [25–27]. However, the accumulation of clinical data regarding the use of the V14, which can be converted for proper single dose equivalence in the future, may support the creation of an ideal calculation method for converting to single dose equivalence from hypofractionation. The V14 obtained to approximate the single dose equivalence is very useful for avoiding adverse effects in hypofraction treatment, in addition to helping identify ideal calculation methods. In any case, the volume of at-risk organs involved within the prescribed isodose should be considered for five-fraction radiotherapy in relation to complications. In the current series, two patients with tumors 21.4 and 33.7 cm^3^ in volume developed radiation necrosis after five-fraction radiotherapy during the early period (between 2006 and 2009). The V14 of these patients ranged from 9.2–19.7 cm^3^. Surgery was performed in both patients to remove radiation necrosis. From 2010 the planned V14 was designed to be < 7.0 cm^3^ to avoid further complications. The V14 may need to be further reduced to < 3 cm^3^ when treating tumors situated deep in the white matter and already exhibiting extensive perifocal edema. Chin *et al*. reported their experiences using a 10 Gy volume during single-fraction radiosurgery based on the Kjellberg 1% risk line and the Flickinger 3% risk line [28]. They reported the median 10 Gy volumes of the normal brain in patients with and without necrosis to be 19.8 and 7.1 cm^3^, respectively.

CyberKnife radiotherapy with a prescribed isodose of 50–60% has the benefits of decreasing the isodose volume (V14) of the surrounding brain in comparison with that in conventional treatment or higher prescription isodose treatment of 80–90% in typical hypofractionation, because a sharp fall-off of the dose distribution is obtained. The rate of radiation necrosis requiring resection was only 2.6% in the patients in this series, including tumors > 30 cm^3^ treated with a mean prescription isodose of 58%. The complication rate is therefore expected to further decrease when the V14 of the surrounding brain is restricted to < 7.0 cm^3^ (applied since 2010), because no radiation necrosis was found in the current series during the later period. The complication data shown in **Fig. 4** are therefore very useful for dose selection in five-fraction radiotherapy. The V14 is one of the important treatment parameters for accurately determining the marginal dose and fraction numbers. For example, when a treatment plan using the prescribed dose of 35 Gy has a V14 > 7.0 cm^3^, the dose should be decreased to 31 Gy. Another method for decreasing the V14 involves decreasing the marginal isodose down to 50% when a treatment plan is made with a marginal isodose of 60–70%. Optimal dose fractionation (multisession radiosurgery) is also possible using the V14, and an increased number of fractions (sessions) is able to decrease the V14 for huge lesions [29].

## CONCLUSION

Five-fraction CyberKnife radiotherapy is safe and effective for patients with large brain metastases in critical areas. An accurate determination of the isodose volume of the surrounding brain is important for decreasing adverse effects, as is also the case in single-fraction radiosurgery. The V14 is a useful indicator of the risk for adverse effects (brain edema and radiation necrosis) in patients with large metastases being treated with five-fraction radiotherapy.
